# Overview of Epidemiology of Bile Duct and Gallbladder Cancer Focusing on the JACC Study

**DOI:** 10.2188/jea.15.S150

**Published:** 2005-08-18

**Authors:** Tsuyoshi Matsuba, Dongmei Qiu, Michiko Kurosawa, Yingsong Lin, Yutaka Inaba, Shogo Kikuchi, Kiyoko Yagyu, Yutaka Motohashi, Akiko Tamakoshi

**Affiliations:** 1 Department of Epidemiology and Environmental Health, Juntendo University School of Medicine.; 2 Department of Public health, Aichi Medical University School of Medicine.; 3 Department of Public Health, Akita University school of Medicine.; 4 Department of Preventive Medicine/ Biostatistics and Medical Decision Making, Nagoya University Graduate School of Medicine.

**Keywords:** Bile Duct Neoplasms, Gallbladder Neoplasms, Epidemiology, the JACC Study

## Abstract

BACKGROUND: This review discusses the epidemiologic features of bile duct and gallbladder cance in Japan, mainly focusing on results of Japan Collaborative Cohort Study (JACC Study) for Evaluation of Cancer Risk Sponsored by the Ministry of Education, Science, Sports and Culture of Japan (Monbusho) in comparison with results of other studies.

METHODS: The theses and papers derived from JACC Study on bile duct and gallbladder cancer were all collected for this review. Hirayama’s cohort study, which is a representative epidemiologic study, and a large scale case-control study on bile duct and gallbladder cancer in Japan by Kato et al. were also taken into consideration. Small scale cross-sectional studies or ecological studies and the studies conducted outside Japan were collected by the literature reference services on the web net such as Pub Med or Japan Centra Revuo Medicina (Igaku- Chuo- Zasshi) limited to the published after 1980 and use key words bile duct cancer, gallbladder cancer and epidemiology.

RESULTS: In the JACC Study, high intake of fried food was regarded as a factor that significantly elevated the risk of the diseases (hazard ratio [HR] = 2.58, 95% confidence interval [CI]: 1.08-6.16 in males; HR = 2.98, 95% CI: 1.28-6.86 in females). The JACC Study indicated that a high intake of boiled beans had a significant preventive relation to the diseases in females (relative risk [RR] = 0.50, 95% CI: 0.26-0.98). High consumption of fish also had a significant preventive relationship to bile duct cancer in males (RR = 0.53, 95% CI: 0.26-0.98) and gallbladder cancer in females (RR = 0.43, 95% CI: 0.24-0.79). A history of blood transfusion also had a significant relationship (HR = 2.27, 95% CI: 1.29-3.98) as which elevated the risk. The JACC Study determined bowel movement as a risk factor. The group with bowel movements less than once per six days had a significantly elevated hazard ratio (HR = 5.21, 95% CI: 1.25-21.68).

CONCLUSION: As to recent epidemiologic features of bile duct and gallbladder cancer revealed by the JACC Study, its outline became obvious in comparison with the results of other studies. Evidence for the contribution of the JACC Study is strong because it provides some important findings on the epidemiology of bile duct and gallbladder cancer.

Recent results of an epidemiologic study of bile duct (without intrahepatic) and gallbladder cancer which are coded in ICD 10th as C23 and C24 in Japan are reviewed. Deaths from these diseases comprise 5% of total cancer deaths in Japan.^1^ As a large scale epidemiologic study in Japan, Japan Collaborative Cohort Study (JACC Study) for Evaluation of Cancer Risk Sponsored by the Ministry of Education, Science, Sports and Culture of Japan (Monbusho)^2^ has reported results of analysis of the relationship of these diseases to nutrition, smoking and drinking alcohol, the habit of drinking coffee, tea and Japanese green tea, history and family history of the diseases, etc. In the subjects Kato et al. conducted a hospital-based case-control study of 193 bile duct and gallbladder cancer cases in Niigata prefecture, known as the mostly high incidence area of the diseases in Japan.^3^ Another analytic epidemiologic study conducted in Japan, which is Hirayama’s cohort study, should be mentioned, which involved domestically the continuous observation of 270,000 subjects from 1966 through 1982. It also provided results on the epidemiology of bile duct and gallbladder cancer.^4^^,^^5^ Moreover, it was available that the series of the small scale cross-sectional studies which treated the environment, especially concerning the effect of the agricultural chemicals in the environment. By and large, here, this review discusses the epidemiologic features of bile duct and gallbladder cancer in Japan mainly through results of the JACC Study in comparison with results of other studies.

## METHODS

Theses and papers reviewed in this article were collected as follows. The JACC Study, as mentioned above, is one of the recent representative cohort studies in Japan, and it produces some theses and papers on bile duct and gallbladder cancer. Those are all included in the review. Hirayama’s cohort study is also a representative epidemiologic study which leads the JACC Study in 1970s and 1980s. As the large scale case-control study on bile duct and gallbladder cancer in Japan, we could only review the thesis by Kato et al. Small scale cross sectional studies or ecological studies and the studies conducted outside Japan were collected by the literature reference services on the web net such as Pub Med or Japan Centra Revuo Medicina (Igaku- Chuo- Zasshi) limited to the published after 1980 and use key words “bile duct cancer”, “gallbladder cancer”, and “epidemiology”.

## RESULTS

In the approximately 12-year follow-up by the JACC Study until 1999, 140 male deaths and 148 female ones (sex ratio 1:1.1) from either bile duct or gallbladder cancer out of a total of 54,032 males and 73,445 females were observed. Deaths from bile duct cancer totaled 91 in males and 74 in females (sex ratio 1:0.8) and those from gallbladder cancer totaled 49 in males and 74 in females (sex ratio 1:1.5). Vital statistics in 2001 in Japan^6^ showed that mortality rates for bile duct and gallbladder cancer were 11.5 per 100,000 population in males and 13.2 in females, with a sex ratio of 1:1.5. Results of the JACC Study was consistent with those of past studies which concluded that bile duct cancer deaths included more males and gallbladder cancer more females.^7^^,^^8^^,^^9^

Table 1 shows peaks in age for deaths from bile duct and gallbladder cancer in the JACC Study. The peak for bile duct cancer was 60-64 years of age in males and 70-74 years in females, and for gallbladder cancer was 70-74 years in males and 75-79 years in females. In both sites, the peak age of death was about 5-10 years older in females. The result showed the same as the vital statistics of Japan in age distribution.

**Table 1.** Age distribution of deaths from bile duct and gallbladder cancer in JACC study.

Site	Sex	Age (year)								

		40-44	45-49	50-54	55-59	60-64	65-69	70-74	75-79	Total
Gallbladder cancer	Male	0(0)	1(2)	1(2)	4(8)	10(20)	16(33)	7(14)	10(20)	49(100)
	Female	1(1)	6(8)	5(7)	9(12)	9(12)	11(15)	22(30)	11(15)	74(100)
Bile duct cancer	Male	1(1)	3(3)	4(4)	19(21)	22(24)	18(20)	14(15)	10(11)	91(100)
	Female	2(3)	3(4)	4(5)	6(8)	11(15)	16(18)	16(18)	16(18)	74(100)

Percentages in parentheses.

The incidence rate of bile duct and gallbladder cancer is extremely high in Chile and Japan globally.^9^^,^^10^^,^^11^ Domestically, in Japan regional accumulation has been detected. Kodama et al. reported that bile duct cancer deaths are more frequent in North East Japan and gallbladder cancer deaths in East Japan both in males and females.^12^ Yamamoto et al. found the result for accumulation of gallbladder cancer cases, with more such deaths reported from hilly areas rather than the plain in Niigata prefecture, Japan.^13^

Kodama et al. detected by secondary medical service areas of Japan where the population is progressively aged that deaths from bile duct and gallbladder cancer had increased.^12^ They pointed out that the population in such areas might be characterized as having a tendency to a traditional lifestyle. A study of Brian et al. in Latin America noted that the incidence of these diseases was higher among the population with a traditional lifestyle distinguished by using language.^14^ It may be challenged as to whether such an analysis should have been conducted in the JACC Study. Although Hirayama analyzed the effect of social levels, which were divided into 4 groups according to occupation, no significant relation was detected.^7^

Table 2 shows the possible relation of food to the risk of bile duct and gallbladder cancer. There was no significant relationship between bile duct and gallbladder cancer and smoking, drinking alcohol, and the habit of taking coffee, tea and Japanese green tea in the JACC Study.^15^^,^^16^ In a case-control study, Kato et al. reported a significant preventive relationship for taking coffee (odds ratio [OR]=0.25, 95% confidence interval [CI]: 0.09-0.68).^3^ Hirayama detected a significant value for the relative risk (RR) of smoking for both bile duct and gallbladder cancers (RR = 1.27, 95% CI: 1.03-1.48)^4^ (Table 2). Zatonski et al. also reported no significant result for a relationship with those items.^17^

**Table 2.** Possible relation of food to the risk of bile duct and gallbladder cancer.

Author	Year	Place	Study design	Site	Item	Sex	Risk ratio	(95% CI)	note
JACC study	1988-1997	Japan	cohort	both	High intake of cheese	male	1.37	(0.71-2.70)	Adjusted risk ratio by using Cox’s proportional hazard model by age Risk ratio of gall bladder cancer in male was not able to be calculatd because of less cases
						female	1.19	(0.61-2.30)	
				both	High intake of butter	male	0.77	(0.40-1.50)	
						female	0.87	(0.46-1.67)	
				both	Moderate intake of fried food	male	2.58	(1.08-6.16)	
						female	2.96	(1.28-6.86)	
				both	Moderate intake of fish	male	0.55	(0.29-1.05)	
						female	0.83	(0.44-1.54)	
				both	High intake of spinach	male	0.99	(0.47-2.07)	
						female	1.73	(0.87-3.43)	
				both	High intake of boiled bean	male	2.09	(0.40-1.51)	
						female	0.67	(0.36-1.21)	
				Gallbladder cancer	High intake of cheese	female	1.26	(0.51-3.09)	
				Gallbladder cancer	High intake of butter	female	1.04	(0.43-2.53)	
				Gallbladder cancer	Moderate intake of fried food	female	3.15	(1.03-9.59)	
				Gallbladder cancer	Moderate intake of fish	female	0.38	(0.16-0.95)	
				Gallbladder cancer	High intake of spinach	female	3.72	(1.38-10.10)	
				Gallbladder cancer	High intake of boiled bean	female	0.79	(0.36-1.71)	
				Bile duct cancer	High intake of cheese	male	1.54	(0.68-3.47)	
						female	1.12	(0.42-2.92)	
				Bile duct cancer	High intake of butter	male	0.88	(0.40-1.97)	
						female	0.72	(0.28-1.81)	
				Bile duct cancer	Moderate intake of fried food	male	1.92	(0.78-4.74)	
						female	2.72	(0.76-9.77)	
				Bile duct cancer	Moderate intake of fish	male	0.74	(0.34-1.61)	
						female	1.76	(0.70-4.43)	
				Bile duct cancer	High intake of spinach	male	1.11	(0.46-2.69)	
						female	0.66	(0.22-1.98)	
				Bile duct cancer	High intake of boiled bean	male	0.47	(0.19-1.15)	
						female	0.55	(0.22-1.35)	

Kato K. et al.	1982-1986	Japan	case-control	Gallbladder cancer	Oily food	both	3.29	(1.68-6.43)	Adjusted odds ratio by using a conditional logis- tic model by foods items.
				Gallbladder cancer	Taking snack (every day)	both	0.26	(0.09-0.72)	
				Gallbladder cancer	Fruit (everyday)	both	0.26	(0.13-0.50)	
				Gallbladder cancer	Coffee (everyday)	both	0.25	(0.09-0.68)	
				Gallbladder cancer	Broiled fish(3 times/week)	both	0.33	(0.15-0.71)	
				Bile duct cancer	Quantity of ingestion (small)	both	3.26	(1.32-8.03)	
				Bile duct cancer	Sliced raw fish (3 times/week)	both	20.60	(1.98-214.11)	
				Bile duct cancer	Alcohol (occasionally-everyday)	both	0.46	(0.23-0.93)	
				Bile duct cancer	Broiled fish (3 times/week)	both	0.39	(0.20-0.75)	

Hirayama T	1966-1982	Japan	cohort	both	Smoking	both	1.27	(1.03-1.48)	Risk ratio not adjusted
				both	Milk (everyday 300 ml/day)	both	1.58	(1.05-2.37)	
				both	Fish (everyday)	both	1.28	(1.11-1.48)	
				Bile duct cancer	Fish (everyday)	both	1.38	(1.12-1.70)	
				Bile duct cancer	Fish (everyday)	female	1.54	(1.12-1.70)	

Serra I, et al.	1992-1995	Chile	case-control	Gallbladder cancer	Fresh fruit (less than 1 piece/week)	both	6.4	(1.4-30.3)	Risk ratio not adjusted
				Gallbladder cancer	Sugar intake as soft drink (rarely)	both	3.6	(1.3-10.1)	
				Gallbladder cancer	Red chili pepper (>20 g/day)	both	2.9	(1.5-5.6)	
				Gallbladder cancer	Green chili pepper (>20 g/day)	both	2.9	(1.3-10.1)	
				Gallbladder cancer	Fried foods (>200 g/day)	both	2.1	(1.1-3.8)	

Pandy M, et al.	2002	India	case-control	Gallbladder cancer	Green chili	both	0.45	(0.21-0.94)	Risk ratio not adjusted
				Gallbladder cancer	Radish	both	0.40	(0.17-0.94)	
				Gallbladder cancer	Sweet potato	both	0.33	(0.13-0.83)	
				Gallbladder cancer	Mango	both	0.40	(0.16-0.99)	
				Gallbladder cancer	Orange	both	0.45	(0.22-0.83)	
				Gallbladder cancer	Melon	both	0.30	(0.14-0.60)	
				Gallbladder cancer	Papaya	both	0.44	(0.20-0.64)	

CI: confidence interval.

Many epidemiologic studies of bile duct and gallbladder cancer have dealt with risk factors related to food and nutrition. Table 2 shows food items for which were revealed significant values for risk ratios in the JACC Study and other recent epidemiologic studies. Table 2 indicates that the JACC Study found that the intake of fried foods significantly elevates the risk of gallbladder and bile duct cancer. Though multivariate analysis did not reveal a significant risk for other food items, the results of univariate analysis indicate the significant relative risks of dairy products such as the intake of cheese (RR = 2.00, 95% CI: 1.13-3.54) and artificial butter (RR = 1.76, 95% CI: 1.00-3.08) for bile duct cancer in males and cheese (RR = 2.23, 95% CI: 1.18-4.22) for gallbladder cancer in females.^1^ Kato et al. reported that the adjusted odds ratio for the liking of the oily foods was significant (OR = 3.29, 95% CI: 1.68-6.43).^3^ Hirayama determined that the relative risk in the group who drank milk everyday (about 300 mL/day) was significantly high (RR = 1.58, 95% CI: 1.05-2.37)(Table 2).^4^ Zatonski et al. reported the no significant relationship between these diseases and intake of fat and dairy products.^18^

As shown in Table 2, the intake of fruit is a significant preventive factor in epidemiologic research in and out of Japan.^3^^,^^7^^,^^8^^,^^10^ The JACC Study, however, also examined the intake of tangerines, citrus fruit and juice, and found no significant relationship. Kato et al. reported that the intake of fruit was associated with a significantly reduced adjusted odds ratio (0.26, 95% CI: 0.13-0.50) for both bile duct and gallbladder cancer (Table 2). A recent large-scale cohort study in Australia showed no significant preventive relationship with the intake of fruit and vegetables, which had been indicated in several cohort studies in the past.^21^ As to consumption of vegetables, the JACC Study revealed that a high intake of spinach elevated the risk of gallbladder cancer in females (hazard ratio [HR] = 3.72, 95% CI: 1.38-10.10).^1^ The results of univariate analysis showed that the moderate intake of cabbage elevated the risk of gallbladder cancer in males (RR = 3.85, 95% CI: 1.13-13.11) and the high intake of boiled beans decreased the risk of both bile duct and gallbladder cancer in females (RR = 0.50, 95% CI: 0.26-0.98).^1^ Pandey et al. reported that in their case-control study, consumption of beans had a tendency to decrease the risk of gallbladder cancer, in spite of the lack of significance.^19^ In the JACC Study, other items such as carrot, tomato, Chinese cabbage, wild plants, mushroom, and potato had no significant relation to the diseases.

Results of univariate analyses in the JACC Study indicated that high intake of fish had a significant preventive relationship with bile duct cancer in males (RR = 0.53, 95% CI: 0.26-0.98) and gallbladder cancer in females (RR = 0.43, 95% CI: 0.24-0.79).^1^ Kato et al. also reported that the adjusted odds ratio in the group who had eaten broiled fish 3 times or more in one week showed a preventive effect (OR = 0.33 95% CI: 0.15-0.71).^3^ Contrary to results of recent studies, Hirayama found the intake of fish (everyday) to be a significant risk factor (RR = 1.28, 95% CI: 1.11-1.48) (Table 2).^4^^,^^5^

Because the intake of chili is rare in Japan, the JACC Study did not address the implications of this food. Concerning results of studies conducted in areas where intake of chili is common, Serra et al. reported significant OR for the intake of green and red chili for gallbladder cancer in their case-control study conducted in Chile.^10^ However, Pandey et al. reported the contrary result that the OR for the consumption of green chili indicated a significant preventive value in India (Table 2).^19^

No significant risk ratios for beef, pork, and chicken intake were revealed in the JACC Study. Kato et al. showed a significant preventive effect by the OR in a group that consumed beef, pork or chicken more than once a week (OR = 0.38, 95% CI: 0.18-0.77) in the result of univariate analysis.^3^ Pandey et al. also suggested consumption of beef or mutton is likely to increase the risk of gallbladder cancer, despite the lack of significance.^19^

Moerman reported that a case-control study showed a relationship between the intake of sugar added to drinks or dessert and gallbladder cancer.^20^ Those factors increased the risk of the disease. However, Serra et al. reported contrarily that the rare intake of soft drinks increases the risk of bile duct and gallbladder cancer (Table 2).^10^

Yamamoto et al. calculated the standardized mortality ratio (SMR) of bile duct and gallbladder cancer for every prefecture in Japan by using data from 1975 and indicated that rates are elevated in North East Japan. It was also revealed that there was a positive relationship between the SMR and the production of rice.^22^ From these results, a role for agricultural chemicals as an environmental factor in these diseases was suspected. Then research was conducted to analyze the ecological correlation between the use of agricultural chemicals (1962-75) and SMR (1975) in 46 prefectures in Japan, and it was concluded that there was a statistically significant relation between the use of 2,4,6-trichlorophenyl-4′-nitrophenylether, 4-chloro-2-methyl-phenoxyacetic, acid and pentachloronitrobenzene and SMR of these diseases.^23^ Other studies conducted to examine the effects of drinking water or soil in Niigata prefecture, Japan, and ecological analyses of the concentration of agricultural chemicals in drinking water and the incidence of the gallbladder cancer suggested a relationship.^24^

About the past history of the cases, previous studies found that histories of cholelithiasis, cholecystitis and typhoid fever were risk factors for bile duct and gallbladder cancer.^3^^,^^10^^,^^11^^,^^18^^,^^26^^,^^27^^,^^28^^,^^29^^,^^30^ The JACC Study found no significant results concerning subject’s and family history of cancer in HRs adjusted by sex and age. A history of blood transfusion showed a significantly high HR to increase the risk of bile duct and gallbladder cancer (HR = 2.27, 95% CI: 1.29-3.98).^30^ Kato et al. reported that in the case of gallbladder cancer, family history of cholelithiasis (OR = 2.96, 95% CI: 1.34-6.50) and history of cholecystitis (OR = 34.36, 95% CI: 4.51-265.96) were regarded as significant risk factors.^3^ Zatonski et al. also reported the positive relationship between such histories and bile duct and gallbladder cancer (Table 3).^17^

**Table 3.** Medical history and bowel movements in relation to risk of bile duct and gallbladder cancer.

Author	Year	Place	Study design	Site	Item	Sex	Risk ratio	(95% CI)	note
JACC study	1988-1997	Japan	cohort	Gallbladder cancer	Bowel movements	both			Adjusted hazard ratio by using Cox’s proportional hazard model by age and sex
					More than once per day		1	(reference)	
					Once per 2 to 5 days		1.48	(0.86-2.54)	
					Less than once per 6 days		5.21	(1.25-21.68)	
Kato K. et al.	1982-1986	Japan	case-control	Gallbladder cancer	Tendency of diarrhea	both			
					Yes		1	(reference)	
					NO		0.48	(0.19-1.20)	
					Do not know		0.22	(0.05-0.88)	

Zatonski et al.	1983-1988	Australia	case-control	Gallbladder cancer	History of cholecystitis	both	34.36	(4.51-265.96)	Risk ratio not adjusted
		Canada		Gallbladder cancer	Family history of cholelithiasis	both	2.96	(1.34-6.50)	
		Netherland		Bile duct cancer	History of cholecystitis	both	12.77	(2.75-59.34)	
		Poland		Bile duct cancer	History of liver diseases	both	27.17	(6.14-120.23)	
				Bile duct cancer	Family history of vascular accident	both	2.23	(1.30-11.68)	

				Gallbladder cancer	History of gallbladder problem	both	4.4	(2.6-7.5)	Adjusted odds ratio by using a conditional logis- tic model by age and sex
				Gallbladder cancer	Surgery for ulcer disease	both	3.0	(1.0-9.4)	
				Gallbladder cancer	Loose stools	both	2.1	(1.2-3.7)	
				Gallbladder cancer	Bowel movements	both			
					Once per day		1.0	(reference)	
					Twice per day		1.6	(0.9-3.1)	
					More than twice per day		8.2	(3.0-22.8)	
					Every second day		1.7	(0.9-3.0)	
					Less frequently than every 2 days		1.7	(0.8-3.6)	

CI: confidence interval.

Table 3 shows that the JACC Study revealed a significant relation between bowel movements and bile duct and gallbladder cancer, namely, that the tendency toward constipation had a role in elevating risk and diarrhea had a role as a preventive factor. Specifically, in the group who had bowel movements less than once per week, the hazard ratio had a significantly high value (HR = 5.21, 95% CI: 1.25-21.68).^28^ Zatonski et al. had considered the factor of bowel movement and their result showed that the tendency toward diarrhea elevated the risk, which is contrary to the result of the JACC Study.^18^

## DISCUSSSION

With regard to distribution of sex, region and socioeconomic factors, the results of the JACC Study and other recent epidemiologic studies in Japan supported those of past epidemiologic studies outside Japan, that is, the morbidity and mortality of gallbladder cancer were greater in females, those were found in some accumulated regions, and those were elevated in the population of the lower socioeconomic areas.^3^^,^^12^ Those findings suggest that risk factors of the diseases can be discovered by epidemiologic studies. Especially, the difference in distribution of the diseases by regions and socioeconomic factors suggests the influence of nutritional, environmental and genetic factors.

Drinking alcohol and smoking, which are often key factors in other cancers and chronic diseases, did not have a significant relation to the diseases examined in the JACC Study^1^. As opposed to the result of the JACC Study, Hirayama had detected a relationship with smoking. Although the smoking rates in Japan at the each time of two studies varied, it is difficult to find out the evidence how the difference of smoking rates affected to the outcome.

The JACC Study also did not show a significant relationship with intake of coffee, tea, and Japanese green tea. However, Kato et al. had reported a significant preventive effect from intake of coffee.^3^ Kato et al. had conducted the study about 10 years earlier than the JACC Study. Thus, because the subjects of the JACC Study were 10 years younger, it might cause the different distribution among the subjects who answered to have the habit of the intake of coffee especially in the peak generation of the cancer deaths. The studies in the United States and Europe, where the proportion of the population consuming coffee has been greater than that of Japan, do not reveal a significant relation with the habit of coffee intake.

About nutrition and foods, the JACC Study noted that consumption of fried foods significantly elevated the risk of those cancer’ death.^1^ Kato et al. also found that the intake of oily foods elevated risk.^3^ On the other hand, among recent epidemiologic studies conducted in the United States and Europe, few cited fat intake as a significant risk factor. Because consumption of fried and oily foods is considered contrary to traditional food habits in Japan, it should be taken into account the confounding factors with socioeconomic factors and the integrated food habit, namely modern or traditional.

Previous studies have reported the preventive effect of the intake of fruits on bile duct and gallbladder cancer; however, the JACC Study did not reveal a significant relationship. A recent study conducted in Australia also reveals the same results as the JACC Study regarding the effect of consumption of fruits.^21^ Reports on the results of other recent studies are expected.

The JACC Study did not indicate a significant preventive relationship for intake of vegetables except boiled beans for bile duct and gallbladder cancer in females being contrary to the results of the other studies conducted up to this time.^1^ High intake of spinach in females and moderate intake of cabbage in males was shown to elevate the risk of gallbladder cancer in JACC Study.^1^

Concerning the intake of fish, the JACC Study concluded that there was a preventive relationship with gall bladder cancer among females and bile duct cancer among males.^1^ Kato et al. also detected a preventive effect of the intake of fish in a case-control study.^3^ Such a discrepancy to the results of Hirayama’s cohort is controversial. More discussion and new findings are necessary to clarify this issue. Some research conducted outside of Japan also reported the effect of intake of fish; however, fish was combined with meats as “intake of protein.” In Japan, it is considered that high intake of fish may be regarded as the tendency toward consumption of traditional foods.

Those findings indicate that traditional food habits may be a preventive factor as a nutritional epidemiologic feature on bile duct and gallbladder cancer in Japan. It appears necessary to perform nutritional analyses to examine the effect of combinations of food items that represent traditional, modern and other diets.

Some studies conducted in countries or regions where the consumption of chili was relatively high reported that its intake elevated risk of those cancers.^9^^,^^10^^,^^11^^,^^19^ However, the JACC Study or other epidemiologic studies in Japan did not consider the intake of chili because the consumption of chili is rare in Japan. It has been reported that the intake of chili is increasing, especially among the young Japanese generation in the context of diversification of food habits. It may be considered as to whether this food item should be included in nutritional epidemiologic studies of bile duct and gallbladder cancer in the future.

As to environmental factors, the series of studies on the relationship between agricultural chemicals and gallbladder cancer by Yamamoto et al. is worthy of special mention as one of the original Japanese epidemiologic studies.^22^^,^^23^^,^^24^

Zatonski et al. detected a relationship between bowel movements and gallbladder cancer, namely, that chronic diarrhea elevated risk.^18^ This was reexamined in the JACC Study and it was concluded that the tendency toward constipation elevated the risk, contrary to the former result.^28^ Perhaps reabsorption of bile caused by bowel movement habits somewhat affected etiological evidence. The role of bowel movement in bile duct and gallbladder cancer should be further examined.

As to recent epidemiologic features of bile duct and gallbladder cancer revealed by the JACC Study, its outline became obvious in comparison with the results of other studies. Evidence for the contribution of the JACC Study is strong because it provides some important findings on the epidemiology of bile duct and gallbladder cancer.

## MEMBER LIST OF THE JACC STUDY GROUP

The present investigators involved, with the co-authorship of this paper, in the JACC Study and their affiliations are as follows: Dr. Akiko Tamakoshi (present chairman of the study group), Nagoya University Graduate School of Medicine; Dr. Mitsuru Mori, Sapporo Medical University School of Medicine; Dr. Yutaka Motohashi, Akita University School of Medicine; Dr. Ichiro Tsuji, Tohoku University Graduate School of Medicine; Dr. Yosikazu Nakamura, Jichi Medical School; Dr. Hiroyasu Iso, Institute of Community Medicine, University of Tsukuba; Dr. Haruo Mikami, Chiba Cancer Center; Dr. Yutaka Inaba, Juntendo University School of Medicine; Dr. Yoshiharu Hoshiyama, University of Human Arts and Sciences; Dr. Hiroshi Suzuki, Niigata University School of Medicine; Dr. Hiroyuki Shimizu, Gifu University School of Medicine; Dr. Hideaki Toyoshima, Nagoya University Graduate School of Medicine; Dr. Kenji Wakai, Aichi Cancer Center Research Institute; Dr. Shinkan Tokudome, Nagoya City University Graduate School of Medical Sciences; Dr. Yoshinori Ito, Fujita Health University School of Health Sciences; Dr. Shuji Hashimoto, Fujita Health University School of Medicine; Dr. Shogo Kikuchi, Aichi Medical University School of Medicine; Dr. Akio Koizumi, Graduate School of Medicine and Faculty of Medicine, Kyoto University; Dr. Takashi Kawamura, Kyoto University Center for Student Health; Dr. Yoshiyuki Watanabe, Kyoto Prefectural University of Medicine Graduate School of Medical Science; Dr. Tsuneharu Miki, Graduate School of Medical Science, Kyoto Prefectural University of Medicine; Dr. Chigusa Date, Faculty of Human Environmental Sciences, Mukogawa Women’s University; Dr. Kiyomi Sakata, Wakayama Medical University; Dr. Takayuki Nose, Tottori University Faculty of Medicine; Dr. Norihiko Hayakawa, Research Institute for Radiation Biology and Medicine, Hiroshima University; Dr. Takesumi Yoshimura, Fukuoka Institute of Health and Environmental Sciences; Dr. Akira Shibata, Kurume University School of Medicine; Dr. Naoyuki Okamoto, Kanagawa Cancer Center; Dr. Hideo Shio, Moriyama Municipal Hospital; Dr. Yoshiyuki Ohno, Asahi Rosai Hospital; Dr. Tomoyuki Kitagawa, Cancer Institute of the Japanese Foundation for Cancer Research; Dr. Toshio Kuroki, Gifu University; and Dr. Kazuo Tajima, Aichi Cancer Center Research Institute.
